# Association of Coronary Collaterals and Myocardial Salvage Measured by Serial Cardiac Magnetic Resonance Imaging after Acute Myocardial Infarction

**DOI:** 10.3390/jcdd10120473

**Published:** 2023-11-24

**Authors:** Jan Pec, Stefan Buchner, Michael Wester, Kurt Debl, Okka W. Hamer, Florian Poschenrieder, Lars S. Maier, Michael Arzt, Stefan Stadler

**Affiliations:** 1 Department of Internal Medicine II, University Hospital Regensburg, 93053 Regensburg, Germany; 2 Department of Internal Medicine, Cham Hospital, 93413 Cham, Germany; 3 Department of Radiology, University Hospital Regensburg, 93053 Regensburg, Germany; 4 Donaustauf Hospital, 93093 Donaustauf, Germany

**Keywords:** myocardial salvage index, coronary collaterals, myocardial infarction, magnetic resonance imaging

## Abstract

Background: Coronary collateral flow in angiography has been linked with lower mortality rates in patients with coronary artery disease. However, the relevance of the underlying mechanism is sparse. Therefore, we tested the hypothesis that in patients with acute myocardial infarction (AMI), relevant coronary collateral flow is associated with more salvaged myocardium and lower risk of developing heart failure. Methods and Results: Patients with first AMI who received a percutaneous coronary intervention within 24 h after symptom onset were classified visually by assigning a Cohen–Rentrop Score (CRS) ranging between 0 (no collaterals) and 3 (complete retrograde filling of the occluded vessel). All 36 patients included in the analysis underwent cardiac magnetic resonance examination within 3 to 5 days after myocardial infarction and after 12 weeks. Patients with relevant collateral flow (CRS 2–3) to the infarct-related artery had significantly smaller final infarct size compared to those without (7 ± 4% vs. 20 ± 12%, *p* < 0.001). In addition, both groups showed improvement in left ventricular ejection fraction early after AMI, whereas the recovery was greater in CRS 2–3 (+8 ± 5% vs. +3 ± 5%, *p* = 0.015). Conclusion: In patients with first AMI, relevant collateral flow to the infarct-related artery was associated with more salvaged myocardium at 12 weeks, translating into greater improvement of systolic left ventricular function. The protective effect of coronary collaterals and the variance of infarct location should be further investigated in larger studies.

## 1. Introduction

Irreversible ischemic myocardial damage is a feared consequence of acute coronary artery occlusion. Immediate percutaneous coronary intervention (PCI) can alleviate the injury of the compromised myocardium, prevent the development of heart failure, and reduce mortality and morbidity [1,2]. Ischemia/reperfusion (I/R) injury during acute myocardial infarction (AMI) results in edema, necrosis formation, microvascular injury, and intramyocardial hemorrhage visible on cardiac magnetic resonance imaging (CMR). T1 weighted images with late gadolinium enhancement (LGE) and T2 weighted images (T2WI) provide important information about myocardium at risk and I/R edema [3]. Importantly, infarct size (LGE) shows negligible temporal dynamics, compared with I/R edema (T2WI), which changes its magnitude rapidly in the first days after AMI [4]. Moreover, neither of the two necessarily equate to necrotic tissue in the first week, demanding additional evaluation of definite scar formation [4]. Therefore, the combination of both modalities with suitable CMR timing might provide a novel insight into dynamic changes of the infarct site.

The occurrence of collateral circulation visible on the angiogram is the result of a process called pruning, in which raised vascular resistance leads to an increase in the caliber of a few large vessels rather than a diminutive increase in the diameter of more smaller vessels [5,6]. In the perfusion territory of the occluded vessel, post-stenotic pressure decreases and longitudinal shear forces in the preformed collateral circulation increase due to flow redistribution. This promotes the remodeling of the vascular supply known as arteriogenesis [6]. Interestingly, in the absence of coronary artery disease, a well-developed collateral function can compensate for up to approximately 18% of the flow and ameliorate symptoms of the briefly occluded vessel [7]. Moreover, angiographically visible collaterals were shown to be associated with smaller final infarct size and improved long-term survival in patients presenting with ST-segment elevation myocardial infarction [8,9]. A meta-analysis had shown an overall 36% reduction in all-cause mortality in patients with coronary artery disease and relevant collateral circulation [10].

Therefore, we tested the hypothesis that in patients with AMI, relevant coronary collateral flow (CRS 2–3) is associated with more salvaged myocardium in serial CMR assessed with T2WI and LGE. In addition, we investigated measures of the severity of heart failure such as left ventricular ejection fraction and NT-proBNP.

## 2. Materials and Methods

### 2.1. Study Population

Patients aged 18 to 80 years admitted to the University Hospital Regensburg (Regensburg, Germany) presenting with a first-time AMI and undergoing immediate PCI within 24 h after symptom onset were prospectively enrolled in the observational study between March 2009 and March 2012. Details of the study design have been published previously [11]. For the purpose of this current retrospective analysis, we evaluated 36 eligible patients. The exclusion criteria were no available CMR (*n* = 19) or angiographic (*n* = 3) data, TIMI flow before PCI ≥ 2 (*n* = 13), and PCI ≥ 24 h (*n* = 3) (Figure S1). 

### 2.2. Coronary Angiography 

All patients underwent primary PCI according to standard clinical practice. All patients were administered aspirin, unfractionated heparin, and P2Y12 inhibitors. 

**Figure 1:**
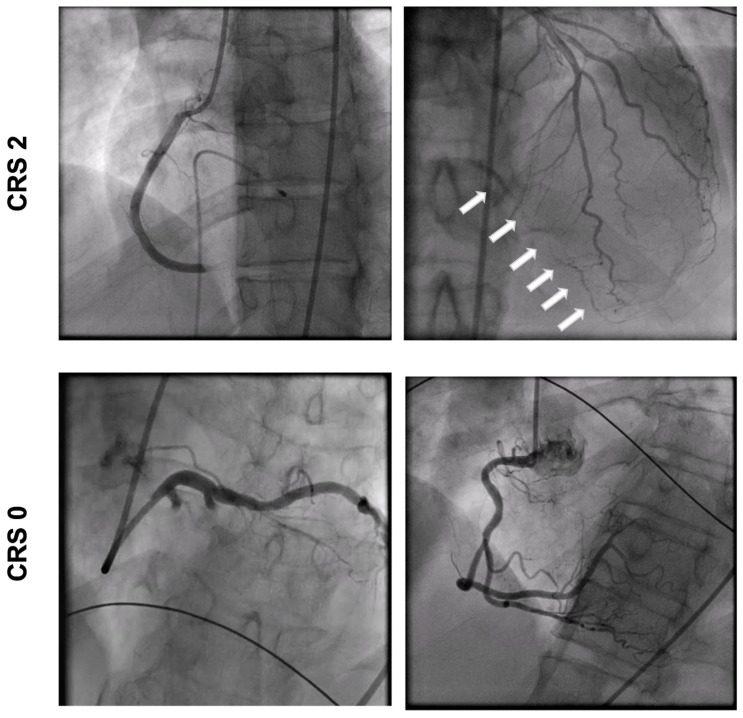
Angiogram of patient with relevant collaterals (CRS 2) and patient with poor collaterals (CRS 0). Arrows: collateral vessels.

Angiograms were assessed by two independent interventional cardiologists blinded to CMR data. The coronary collateral flow was estimated visually using the Cohen–Rentrop Score (CRS): grade 0, no filling of collateral vessels; grade 1, filling of collateral vessels without any opacification of epicardial recipient artery; grade 2, partial filling of target epicardial artery by collateral vessels; and grade 3, complete epicardial filling of recipient artery by collaterals [12] (Figure 1). The infarct-related artery and culprit lesion were identified based on the loss of antegrade flow and the change in flow after PCI. The antegrade flow was assessed using the TIMI classification of perfusion: grade 0, no perfusion; grade 1, penetration without perfusion; grade 2, partial perfusion; and grade 3, complete perfusion. The intraclass correlation coefficient indicated good reproducibility of the CRS assessment between two observers (ICC = 0.872, *p* < 0.001) [13]. 

### 2.3. Cardiovascular Magnetic Resonance

Details of CMR data acquisition are described previously [11,14]. Eligible patients received CMR within 3–5 days and 12 weeks after PCI. CMR was performed on a clinical 1.5 Tesla scanner (Avanto, Siemens Healthcare Sector, Erlangen, Germany) using a phased array receiver coil during breath-hold and was ECG-triggered. Examination of ventricular function was performed by the acquisition of steady-state free precession (SSFP) cine images in standard short-axis planes (trueFISP). For short-axis T2w-STIR imaging, a breath-hold black-blood turbo spin echo technique was adopted using a triple inversion recovery preparation module. Finally, short-axis delayed enhancement images were obtained by use of a segmented inversion recovery SSFP technique and acquired 10–15 min after injection of Gadovist.

Calculation of left ventricular volumes and left ventricular ejection fraction was performed in the serial short-axis slices using commercially available software (syngo Argus, version B15; Siemens Healthcare Sector). The extent of edematous myocardium and delayed enhancement in each image was quantified with custom analysis software (VPT, Siemens Corporate Research, Princeton, NJ, USA) [15]. After manual tracing of pericardial and endocardial contours, a region of interest was drawn within a remote non-infarcted myocardium segment. On delayed enhancement imaging, myocardial infarction was considered to be present if the signal intensity of hyper-enhanced myocardium was greater than five standard deviations (SDs) above the mean signal intensity of the remote region [16]. On T2WI, I/R edema was considered present if the signal intensity of the myocardium was greater than two SDs above the mean signal intensity of the remote non-infarcted myocardium region [17,18].

MSI was calculated as the difference between I/R edema (% left ventricular mass (LVM); baseline T2WI) and final infarct size (% LVM; follow-up LGE) divided by I/R edema (% LVM; baseline T2WI).

### 2.4. Statistical Analysis 

After testing for normality using the Shapiro–Wilk test, continuous variables were compared using Student’s *t*-test or Welch’s test according to Levene’s test. A Mann–Whitney U test was calculated to compare differences between two independent groups when the dependent variable was not normally distributed. For categorical variables, the Chi-square or Fisher’s exact test was used depending on the number of observations. Descriptive data are expressed as means ± standard deviation (SD), median with interquartile range (IQR), or frequencies and percentages of each category. Paired *t*-tests were performed to compare the results of initial and repeated measurements. An exploratory sensitivity analysis was performed to account for the main limitations of the study. Patients presenting exclusively with STEMI, TIMI flow before PCI 0–3, and a culprit lesion in the right coronary artery were compared, respectively, based on the CRS using Student’s *t*-test. The intraclass correlation coefficient (SPSS two-way mixed model, type absolute agreement) was used to assess the reproducibility of CRS. All reported *p* values are two-sided and the threshold for significance was set at *p* < 0.05. Statistical analysis was performed in SPSS (SPSS Statistics for Mac OS, Version 29.0 Armonk, NY, USA: IBM Corp.) and GraphPad Prism (Version 9.51 for Windows, GraphPad Software, La Jolla, CA, USA).

## 3. Results

A total of 36 patients (mean age 56 ± 9 years; 81% men) were included in this analysis. Most patients presented with ST-elevation myocardial infarction (86.1%). The median time from symptom onset to revascularization was 234 min (170–487 min). A total of 19 (52.8%) patients were treated with thrombus aspiration, and 31 (86.1%) patients received glycoprotein IIb/IIIa inhibitors during the procedure as decided by the interventional cardiologist (Table 1). Patients were stratified into two groups based on the CRS according to the degree of collateral circulation: CRS 0–1, poor collateral flow, and CRS 2–3, relevant collateral flow. There were no significant differences in demographic parameters or comorbidities between both groups (Table 1). In patients with CRS 2–3, the culprit lesion was found exclusively in the right coronary artery, whereas in the group with poor collateralization, the culprit lesion was mostly found in the left anterior descending artery. There were no differences in the time to revascularization after symptom onset, ST deviation on ECG, or laboratory parameters (Table 1). 

**Figure 2:**
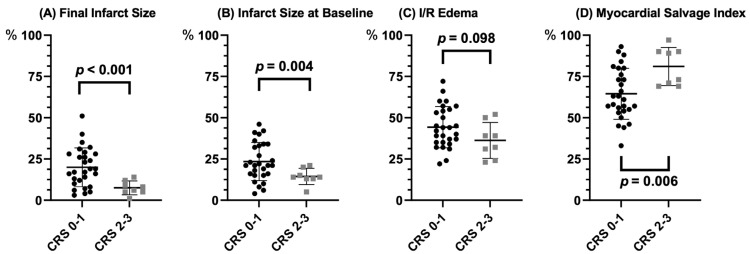
(**A**) final infarct size; (**B**) infarct size at baseline; (**C**) I/R edema; (**D**) myocardial salvage index according to Cohen–Rentrop Score (CRS 0–1: black; CRS 2–3: grey). I/R: ischemia/reperfusion.

Infarct size at baseline and week 12 were significantly smaller in patients with CRS 2–3 (Figure 2A,B, Table 2). I/R edema was similar between the CRS 0–1 and CRS 2–3 groups (Figure 2C, Table 2). The presence of relevant collateral flow was also significantly associated with higher MSI (Figure 2D, Table 2). The reduction in infarct size from baseline to 12 weeks was numerically greater in CRS 2–3 vs. CRS 0–1 (Table 2). Infarct size decreased in all patients significantly (−4.3%, CI −5.9 to −2.6%, *p* < 0.001).

**Figure 3:**
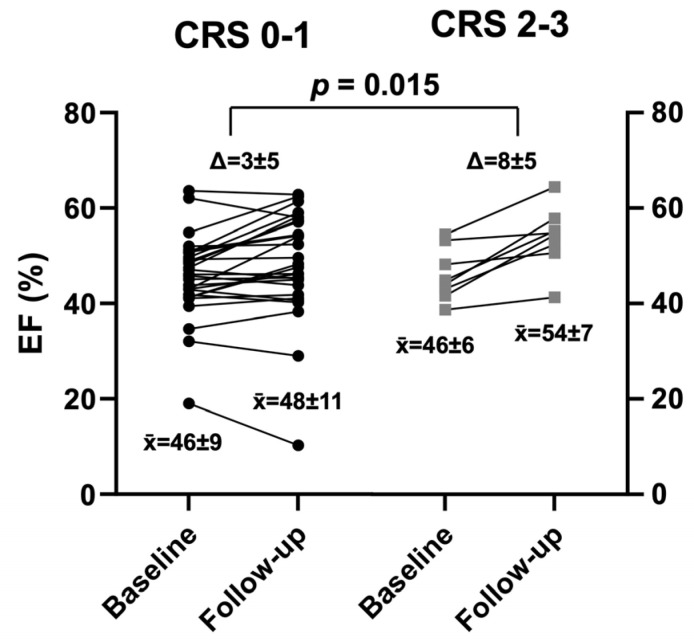
Left ventricular ejection fraction (EF) at baseline and follow-up according to Cohen–Rentrop Score (CRS 0–1: black: CRS 2–3: grey). Δ: change; $\bar{x}$ mean.

In addition, both groups showed improvement in left ventricular ejection fraction early after AMI (Figure 3, Table 2), whereas the recovery was greater in CRS 2–3 compared to those with CRS 0–1 (+8 ± 5% vs. +3 ± 5%, *p* = 0.015).

NT-proBNP decreased in 12 weeks numerically more in patients with CRS 2–3 −616 pg/mL [213–1547] vs. −384 pg/mL [231–709], *p* = 0.313) (Figure S2). Clinical parameters for classifying the severity of heart failure (NYHA) or clinical score for angina severity (CCS) showed no significant differences between both groups (Table S1). 

Volumetric parameters such as left ventricular end-diastolic volume or end-systolic volume did not change significantly (Table S2). 

Even after the inclusion of patients with a TIMI flow ≥ 2, there was a significant association of CRS 2–3 with smaller final infarct size (*p* = 0.013). Similarly, a statistically significant association of CRS 2–3 with smaller final infarct size was observed after the exclusion of patients presenting with NSTEMI (*p* < 0.001). To account for the unequal distribution of infarct location, a sensitivity analysis was performed including patients (CRS 0–1 *n* = 5; CRS 2–3 *n* = 8) with a culprit lesion exclusively in the right coronary artery, which showed a strong association of CRS 2–3 with smaller final infarct size (*p* = 0.052). 

## 4. Discussion

**Figure 4:**
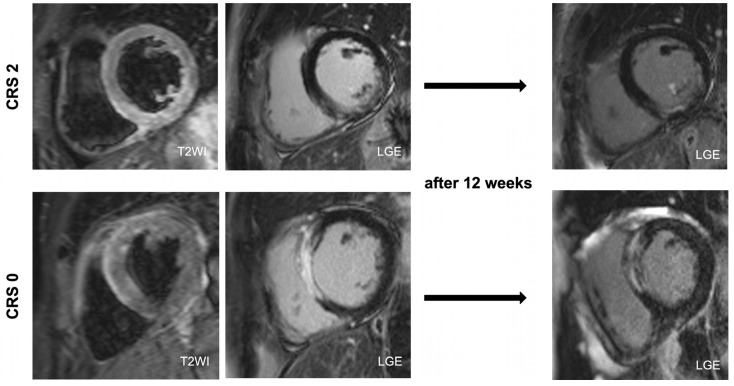
Patient with CRS 2 (I/R Edema 52%; IS at baseline 14%; FIS 5%; MSI 90%) and patient with CRS 0 (I/R Edema 60%; IS at baseline 46%; FIS 51%; MSI 15%). CRS: Cohen–Rentrop Score; FIS: final infarct size; I/R: ischemia/reperfusion; IS: infarct size.

This study evaluated the protective role of coronary collaterals early after AMI. Firstly, we observed smaller final infarct size in CRS 2–3 at 12 weeks (Figure 4). Secondly, CRS 2–3 was accompanied by improved left ventricular ejection function indicating a lower probability of developing heart failure. Thus, our results contribute to the existing evidence of a cardioprotective effect of collateral circulation on myocardial salvage after AMI. 

According to previous studies, there is growing evidence about the protective role of collateral flow in patients with AMI. However, some of these studies were based on only one CMR at baseline [8,19,20,21] or used exclusively LGE to determine myocardial damage [22,23]. Our study is the first to assess I/R edema within 3–5 days after AMI with T2WI in addition to LGE at baseline and 12 weeks after AMI. Fernández-Jiménez et al., demonstrated in a porcine model that post-MI edema formation measured with CMR altered rapidly early after AMI. At first, intense extracellular edema formation occurring 120 min after I/R injury diminished within the next 24 h, and then appeared again with a stable dynamic within days 4 and 7. Secondly, they showed that myocardial edema was greatly influenced by the application of preconditioning with corresponding tissue composition changes. Interestingly, they reported similar I/R edema with T2W-STIR in I/R protocol with and without preconditioning, but a great difference in infarct size assessed with LGE at day 4 [4]. Our results reflect those observations. Infarct size at baseline was significantly smaller in CRS 2–3 but I/R edema was similar at the same time. This finding suggests that the presence of good coronary collaterals depicts viable vessels capable of arteriogenesis and thus acting in a similar way as preconditioning. Moreover, the fact that CRS 2–3 was associated with smaller infarct size assessed with LGE at baseline as well as at follow-up might implicate that LGE extent is more robust to depict the “dead” myocardium and predict final infarct size. The overall infarct shrinkage can be more attributed to partial volume effect due to islands of necrosis at the infarct border that later involute [3]. 

In light of the dynamic changes in I/R edema, calculating for MSI is challenging and currently not recommended as a primary CMR endpoint [3]. Nevertheless, the assessment of MSI based on I/R edema measured within 3–5 days after AMI in the present study showed conclusive results. As noted previously, I/R edema was found to change rapidly during the first two days, and thus could yield misleading results [3]. 

Another important finding of this study was a significantly improved left ventricular function associated with CRS 2–3, which was consistent with a previous study [22]. Interestingly, NT-proBNP decreased more in the group with CRS 2–3 after 12 weeks, whereas both left ventricular ejection fraction and NT-proBNP are diagnostic parameters for heart failure. These results suggest a possible beneficial effect of collateral flow on the development of heart failure, which may project into lower rates of mortality or hospitalization. Our data could not determine whether there was also better symptom control, as NYHA or CCS showed no relevant difference between both groups. A longer follow-up would perhaps provide more information. 

Regarding the survival benefit [9] and smaller infarct size of patients with good collateral circulation, its therapeutic promotion appears as a hopeful target to salvage the myocardium in acute coronary artery occlusion. However, any biochemical concepts of induction of angiogenesis or arteriogenesis with angiogenic growth factors, or biophysical concepts failed to prove any relevant clinical effect or were prone to potentially harmful effects [24]. Therefore, future research on alternative therapeutic promotion of collaterals is needed as there may be a beneficial effect on ischemic and reperfusion injury. 

Some limitations need attention. Firstly, statistical power is limited by the relatively small sample of patients included in this analysis. In addition, in patients with relevant collaterals, the culprit lesion was located exclusively in the right coronary artery. In contrast, the existing evidence reported on an equal distribution of the culprit lesion in both CRS groups and hence may represent a selection bias. Furthermore, despite the relatively young age of the patients, we included patients with pre-infarction angina and patients with multivessel disease indicative of chronic coronary artery disease, which may potentially correspond to a different stage of collateral remodeling. Additionally, most patients in this analysis were treated with GP IIb/IIIa inhibitors during PCI or received thrombus aspiration currently not routinely recommended [25], limiting the generalizability on current patients. Finally, the grading of collateral flow is subjective and prone to inter-observer variance. 

## 5. Conclusions

In patients with the first AMI, relevant collateral flow to the infarct-related artery was associated with smaller final infarct size at 12 weeks, translating into greater improvement of systolic left ventricular function. The protective effect of coronary collaterals and the variance in infarct location should be further investigated in larger studies.

## Figures and Tables

**Table 1:** Baseline Characteristics.

	All Patients	CRS 0–1	CRS 2–3	
	(*n* = 36)	(*n* = 28)	(*n* = 8)	*p* Value
Age, years	56 (±9)	55 (±9)	57 (±10)	0.57
Male gender, *n* (%)	29 (80.6)	24 (86)	5 (63)	0.17
BMI, kg/m^2^	28.1 (±3.4)	29 (±3)	27 (±3)	0.16
Systolic blood pressure, mmHg	128 (±23)	128 (±25)	128 (±18)	0.99
Diastolic blood pressure, mmHg	79 (±13)	80 (±14)	77 (±7)	0.55
Heart rate, bpm	67 (59–78)	67 (59–78)	68 (63–77)	0.76
Arterial hypertension, *n* (%)	19 (53)	16 (57)	3 (38)	0.43
Diabetes mellitus, *n* (%)	6 (17)	5 (18)	1 (13)	1.00
History of smoking, *n* (%)	26 (72)	20 (71)	6 (75)	1.00
Pre-infarction angina (CCS I-IV), *n* (%)	6 (16.7)	4 (17)	2 (33)	0.58
AHI,/h	8 (4–23)	14 (4–28)	6 (4–9)	0.20
Troponin I before PCI, ng/mL	2.54 (0.03–17.25)	2.66 (0.02–23.25)	2.53 (0.05–5.54)	0.90
CK-MB max, U/L	37 (±21)	38 (±22)	31 (±4)	0.60
NT-proBNP max, pg/mL	897 (497–1406)	721 (240–1239)	897 (505–1442)	0.28
LDL, mg/dL	117 (±24)	119 (±22)	111 (±32)	0.38
Creatinine, mg/dL	0.98 (±0.25)	0.89 (±0.21)	1.06 (±0.54)	0.40
eGFR, mL/min/1.73 m^2^	89 (±19)	92 (±16)	78 (±26)	0.07
CRP, mg/L	4.4 (1.9–13.5)	4.8 (2.4–14.7)	2 (0.9–9.8)	0.16
STEMI, *n* (%)	31 (86)	24 (86)	7 (86)	1.00
Multivessel disease, *n* (%)	15 (42)	10 (36)	5 (63)	0.24
Chronic total occlusion, *n* (%)	0 (0)	0 (0)	0 (0)	1.00
Culprit vessel				<0.001
LAD, *n* (%)	14 (39)	14 (50)	0	
LCX, *n* (%)	9 (25)	9 (32)	0	
RCA, *n* (%)	13 (36)	5 (18)	8 (100)	
Culprit lesion location				0.59
Proximal, *n* (%)	17 (47)	12 (43)	5 (62.5)	
Medial, *n* (%)	13 (36)	11 (39)	2 (25)	
Distal, *n* (%)	3 (8)	2 (7)	1 (12.5)	
Side branch, *n* (%)	3 (8)	3 (11)	0	
TIMI flow before PCI				0.57
TIMI 0, *n* (%)	31 (86)	23 (82)	8 (100)	
TIMI 1, *n* (%)	5 (14)	5 (18)	0	
TIMI flow after PCI				1.00
TIMI 2, *n* (%)	3 (8)	3 (11)	0	
TIMI 3, *n* (%)	33 (92)	25 (89)	8 (100)	
Pain-to-balloon time, min	234 (170–487)	234 (170–603)	254 (167–381)	0.72
ST deviation, mV	0.11 (0.08–0.17)	0.12 (0.08–0.19)	0.09 (0.04–0.13)	0.15
Glycoprotein IIb/IIIa inhibitor, *n* (%)	31 (86)	23 (82)	8 (100)	0.57
Thrombus aspiration, *n* (%)	19 (53)	15 (54)	4 (50)	1.00

AHI: apnea-hypopnea-index; BMI: body mass index; CK-MB: creatine kinase–myocardial band; CRP: c-reactive protein; CRS: Cohen–Rentrop Score; eGFR: estimated glomerular filtration rate; LAD: left anterior descending artery; LCX: left circumflex artery; LDL: low density protein; NT-proBNP: N-terminal pro-B-type natriuretic peptide; RCA: right coronary artery; STEMI: ST-elevation myocardial infarction. Values are expressed as means ±95 standard deviation (SD), median with interquartile range (IQR), or frequencies and percentages of each category. Bold values mean statistical significance.

**Table 2:** Cardiac Magnetic Resonance Imaging.

	CRS 0–1	CRS 2–3	
	(*n* = 28)	(*n* = 8)	*p* Value
Infarct size at baseline, %	23 (±11)	14 (±5)	0.004
Final infarct size, %	20 (±12)	7 (±4)	<0.001
I/R edema at baseline, %	44 (±12)	36 (±11)	0.098
Myocardial salvage index, %	57 (±19)	78 (14)	0.006
Left ventricular ejection fraction			
EF at baseline, %	46 (±9)	46 (±6)	0.89
EF at follow-up, %	48 (±11)	54 (±7)	0.18
Difference between baseline and follow-up			
Infarct size, %	−3 (±5)	−7 (±5)	0.051
EF, %	3 (±5)	8 (±5)	0.015

CRS: Cohen–Rentrop Score; EF: ejection fraction; I/R: ischemia/reperfusion. Values are expressed as means ±95 standard deviation (SD). Bold values mean statistical significance.

## Data Availability

The data underlying this article will be shared on reasonable request to the corresponding author. The data are not publicly available due to privacy restrictions.

## Supplementary Materials

The following supporting information can be downloaded at: https://www.mdpi.com/article/10.3390/jcdd10120473/s1, Figure S1: Flow diagram of patients included in the study; Figure S2: NT-proBNP at baseline and follow-up according to Cohen–Rentrop Score.; Table S1: Clinical Parameters; Table S2: Volumetric CMR Parameters According to Cohen–Rentrop Score; Table S3: Medication at discharge and follow-up.
