# Supplementary material for: Association of Coronary Collaterals and Myocardial Salvage Measured by Serial Cardiac Magnetic Resonance Imaging after Acute Myocardial Infarction
Source: J Cardiovasc Dev Dis. 2023 Nov 24;10(12):473. doi: 10.3390/jcdd10120473 (PMC10743778; doi:10.3390/jcdd10120473)
Supplement: Supplementary file 1 [file jcdd-10-00473-s001.zip › jcdd-2697183-supplementary.pdf]

Supplement:

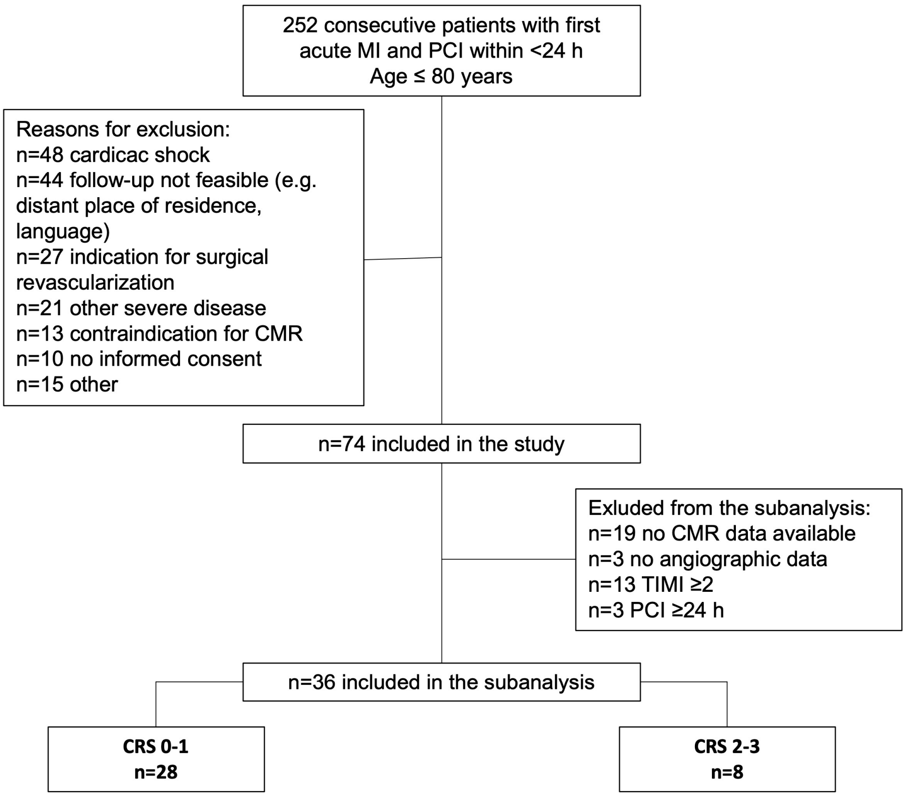

Figure S1. Flow diagram of patients included in the study. CAD: coronary artery disease; CMR: cardiac magnetic resonance; CRS: Cohen-Rentrop Score; MI: myocardial infarction; PCI: percutaneous coronary intervention.

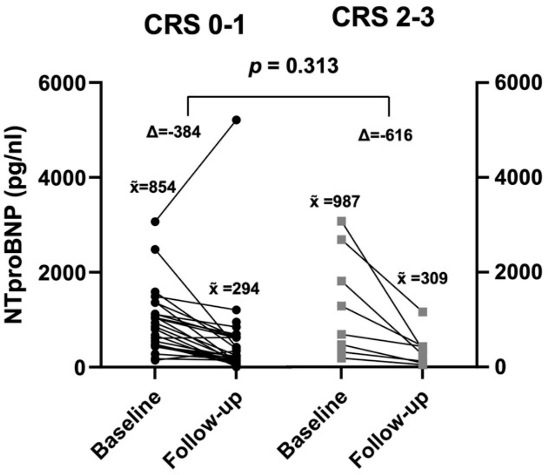

Figure S2. NT-proBNP at baseline and follow-up according to Cohen-Rentrop Score (CRS 0-1: black: CRS 2-3: grey). Δ: change; x̃: median.

Table S1. Clinical Parameters.

New York Heart Association (NYHA)	**CRS 0-1** (n = 28)	**CRS 2-3** (n = 8)	p Value
Baseline			
Class I	19 (67.9%)	4 (50%)	
Class II	6 (21.4%)	3 (37.5%)	
Class III	0	0	
Class IV	0	0	
Follow-up			
Class I	19 (67.9%)	6 (75%)	
Class II	4 (14.3%)	1 (12.5%)	
Class III	1 (3.6%)	0	
Class IV	0	0	
Difference between baseline and follow-up			0.552
No change	17 (60.7%)	5 (62.5%)	
Worse (Δ +1)	2 (7.1%)	1 (12.5%)	
Better (Δ -1)	3 (10.7%)	0	
Canadian Cardiovascular Society (CCS)			
Baseline			
Class 0	19 (67.9%)	4 (50%)	
Class I	4 (14.3%)	2 (25%)	
Class II	1 (3.6%)	1 (12.5%)	
Class III	0	0	
Class IV	0	0	
Follow-up			
Class 0	18 (64.3%)	7 (87.5%)	
Class I	5 (17.9%)	0	
Class II	1 (3.6%)	0	
Class III	0	0	
Class IV	1 (3.6%)	0	
Difference between baseline and follow-up			0.118
No change	16 (57.1%)	4 (50%)	
Worse (Δ +1)	2 (7.1%)	0	
Better (Δ -1)	4 (14.3%)	2 (25%)	
Better (Δ -2)	0	1 (12.5%)	

CRS: Cohen-Rentrop Score; Values are expressed as frequencies and percentages of each category.

Table S2. Volumetric CMR Parameters According to Cohen-Rentrop Score.

Baseline	**CRS 0-1** (n = 28)	**CRS 2-3** (n = 8)	p Value
LVEDV, ml/m²	79 (±12)	76 (±17)	0.555
LVESV, ml/m²	43 (±12)	41 (±11)	0.656
LVM, g/m²	75 (±11)	65 (±17)	0.057
Follow-up after 12 weeks			
LVEDV, ml/m²	81 (±13)	62 (±24)	0.083
LVESV, ml/m²	43 (±13)	34 (±12)	0.126
LVM, g/m²	65 (±10)	61 (±12)	0.345

Difference between baseline and follow-up			
LVEDV, ml/m²	2 (±13)	-14 (±31)	0.195
LVESV, ml/m²	1 (±7)	-7 (±9)	0.057

CRS: Cohen-Rentrop Score; CMR: cardiac magnetic resonance; LVEDV: left ventricular end-diastolic volume; LVESV: left ventricular end-systolic volume; LVM: left ventricular mass. Values are expressed as means ±95 standard deviation (SD). Bold values mean statistical significance.

Table S3. Medication at discharge and follow-up.

At discharge	CRS 0-1 (*n* = 28)	CRS 2-3 (*n* = 8)	*p* Value
ACEi/ARB at discharge, n (%)	27 (96)	8 (100)	1.00
β-Blocker at discharge, n (%)	27 (96)	8 (100)	1.00
Loop diuretics at discharge, n (%)	10 (36)	2 (25)	0.69
MRA at discharge, n (%)	19 (68)	2 (25)	**0.046**
P2Y12 inhibitor, n (%)	28 (100)	28 (100)	1.00
Statin, n (%)	26 (93)	8 (100)	1.00
**At follow-up**			
ACEi/ARB, n (%)	25 (100)	8 (100)	1.00
β-Blocker, n (%)	25 (96)	7 (88)	0.42
Loop diuretics, n (%)	12 (46)	2 (29)	0.67
MRA, n (%)	12 (46)	2 (25)	0.42
P2Y12 inhibitor, n (%)	18 (100)	8 (100)	1.00
Statin, n (%)	26 (100)	8 (100)	1.00

ACEi: ACE-inhibitor; ARB: angiotensin receptor blocker; CRS: Cohen-Rentrop Score; MRA: Mineralocorticoid receptor antagonist. Values are expressed as means ±95 standard deviation (SD). Bold values mean statistical significance.
